# Visual Dysfunction in Posterior Cortical Atrophy

**DOI:** 10.3389/fneur.2017.00389

**Published:** 2017-08-16

**Authors:** Mari N. Maia da Silva, Rebecca S. Millington, Holly Bridge, Merle James-Galton, Gordon T. Plant

**Affiliations:** ^1^The National Hospital for Neurology and Neurosurgery, London, United Kingdom; ^2^Cognitive and Behavioural Neurology Unit, Hospital das Clínicas, University of São Paulo, São Paulo, Brazil; ^3^Oxford Centre for fMRI of the Brain (FMRIB), Nuffield Department of Clinical Neurosciences, University of Oxford, Oxford, United Kingdom; ^4^Moorfields Eye Hospital, London, United Kingdom; ^5^St. Thomas’ Hospital, London, United Kingdom

**Keywords:** posterior cortical atrophy, Alzheimer’s disease (AD), Balint’s syndrome, visual agnosia, visual fields, hemianopia, magnetic resonance imaging imaging

## Abstract

Posterior cortical atrophy (PCA) is a syndromic diagnosis. It is characterized by progressive impairment of higher (cortical) visual function with imaging evidence of degeneration affecting the occipital, parietal, and posterior temporal lobes bilaterally. Most cases will prove to have Alzheimer pathology. The aim of this review is to summarize the development of the concept of this disorder since it was first introduced. A critical discussion of the evolving diagnostic criteria is presented and the differential diagnosis with regard to the underlying pathology is reviewed. Emphasis is given to the visual dysfunction that defines the disorder, and the classical deficits, such as simultanagnosia and visual agnosia, as well as the more recently recognized visual field defects, are reviewed, along with the evidence on their neural correlates. The latest developments on the imaging of PCA are summarized, with special attention to its role on the differential diagnosis with related conditions.

## Introduction

In 1988, Benson et al. described an intriguing progressive condition characterized by a complex visual disorder occurring in the absence of ocular dysfunction (1). Their title introduced the syndromic diagnosis “posterior cortical atrophy” (PCA) which has proven apt and has survived to the present. The most common deficits in their cohort were components of the Bálint (simultanagnosia, optic ataxia, and ocular apraxia) and Gerstmann (agraphia, acalculia, finger agnosia, and right-left disorientation) syndromes (2, 3); additional features were alexia, visual agnosia, and transcortical sensory aphasia. Eventually non-visual functions, such as language and memory, were affected but until a very late stage these deficits were relatively mild and the visual disorder remained the main source of impairment throughout the course of the disease. Insight was preserved until late. Neuroimaging of these patients indicated disproportionate volume loss in the posterior cortical regions, particularly in the occipital and posterior parietal lobes (1). This clinicoradiological syndrome was soon found to be associated with AD (4, 5), but, as opposed to the typical (amnestic) form of the disease, there was an anterior–posterior gradient in PCA, with the greater severity of change occurring in the occipital, parietal and posterior temporal lobes.

With an increasing number of cases coming to autopsy other pathological entities—such as corticobasal degeneration (CBD), Lewy body disease (LBD), and prion diseases (6, 7)—were occasionally described as the underlying cause of PCA. More recently, PCA was reported in an individual with a mutation associated with frontotemporal dementia (8). AD, however, remains the single dominant cause, accounting for 62–100% of the cases in the largest cohorts (6, 7, 9). Thus, PCA has been recognized as one of the atypical variants of AD (10) and indeed it is occasionally referred to as the “visual variant” of AD but this implies a more certain pathological diagnosis than is usually the case. From an integrative perspective, it may also be understood within a continuum of phenotypic variation of AD, since considerable clinical overlap occurs between PCA and other AD variants, especially its amnestic and language presentations (11, 12), more so at later stages. With time all PCA patients will progress to dementia of the AD type. However, it is as a visual disorder that PCA gains singularity, for the visual deficits are usually extremely disabling, even when the patient may still be considered cognitively preserved. This characteristic implies that PCA patients differ from patients with more cognitive presentations of AD in several aspects, including diagnosis and management.

## Epidemiology

The prevalence of PCA is unknown. In clinical cohorts published by centers for cognitive disorders, it has been found to represent about 5% of the total AD cases (13, 14). No estimate is available from ophthalmic services. Age at diagnosis is mostly within the late 50s and early 60s (15–17) but PCA can affect individuals from the 40s (18) to the 80s (7). Females and males are equally represented in several studies (7, 17), but some have observed a female predominance (18–20).

Patients with PCA and their families will usually describe a time-consuming search for the diagnosis, including appointments with several specialists—usually optometrists and ophthalmologists—before a neurological disorder is suspected (21). It is not rare for patients to be provided with numerous pairs of spectacles or even undergo cataract surgery or other procedures only to learn later that the problem is not in the eyes. Given this experience, it is a common impression amongst specialists dealing with PCA patients that this condition is underdiagnosed.

## Clinical Profile

### Visual Manifestations

One of the difficulties in diagnosing patients with PCA is that, although they complain about problems with their vision, descriptions of their symptoms are often difficult for the non-specialist to analyze. They may just say they cannot see, describe their vision as blurry, or may refer to difficulties performing specific tasks such as driving or reading. Only a detailed examination may uncover the specific deficit(s) leading to functional impairment. In the following sections the most important visual deficits in PCA are described.

### Simultanagnosia

Simultanagnosia refers to the failure to perceive multiple visual locations simultaneously or to shift attention from one object to another, which results in a very restricted effective visual field (22). A patient may miss an object he or she has just seen or report that objects seem to appear or disappear from view. Simultanagnosia has been consistently demonstrated as the most frequent deficit in PCA, occurring in above 90% of the patients in several series (7, 15–17). It is a pervasive deficit that may be associated with some unusual behavior including the reverse-size phenomenon. This describes patients preferring to look at objects at distance, in order to appreciate them globally, or finding it easier to read small than large letters—such as the text rather than the headlines of a newspaper (23). In severe cases of simultanagnosia, perception of even a single, large object may be impaired, and sometimes an individual part of it may be mistaken for a different object [so-called “partonomic” error (24)].

Tasks relying on visual integration are used to test for simultanagnosia. Established tests include interpretation of a complex visual scene (25), such as the Boston cookie-theft picture, and reading fragmented letters. Failure to read the Ishihara pseudoisochromatic plates despite preserved color perception is a conspicuous feature in many patients with simultanagnosia (24, 26). The latter is often the only abnormality seen in the basic visual assessment of a PCA patient and its usefulness to raise the diagnostic suspicion cannot be overemphasized, although it is usually misinterpreted as a color deficit. However, the patients have as much trouble with the first (control) plate, which does not require color vision as do the subsequent plates. In its purest form simultanagnosia is considered due to impaired visual attention, which can be considered both in terms of shifts of attention to regions within the visual field and also shifts of attention related to the scale of the object to be processed. The former will be mirrored in impairment of ocular motor behavior but the latter may not be. However, it has also been argued that the attentional deficit may be object based resulting in failure to identify overlapping figures (objects at the same spatial location), or collocated objects where linking features have been weakened [such identifying correctly a star of David where the two component triangles are the same but not different colors (27)]. It should also be considered that the perception of illusory contours is a very early process in object identification and may occur as early as V2: this is likely related to the synthesis of partially occluded objects (28). Impairment of this early function in the identification of surfaces, which has been reported in simultanagnosia (29) would certainly seriously impair the identification of fragmented images.

### Other Elements of the Bálint Syndrome—Optic Ataxia and Ocular Apraxia

Simultanagnosia may occur in isolation or may be associated with optic ataxia and ocular apraxia, constituting the Bálint syndrome. Optic ataxia—lack of eye-hand coordination—refers to impaired reaching to objects when guided by vision with the preserved ability to do so when the object is accessed by means of other sensory modalities, e.g., sound (22), while ocular apraxia is a disorder of fixation, with the patient failing to fixate a specific object within the visual field in the absence of any ocular motor deficit (30). In PCA, the Bálint syndrome is often incomplete. Silmultanagnosia is thought to be an early finding, initially presenting in isolation or associated with ocular apraxia, with optic ataxia developing later in the course of disease (17). Bálint syndrome is classically seen in the context of biparietal damage due to vascular disorders; however, it has been associated with PCA so often that its occurrence in a progressive manner should raise suspicion of the diagnosis.

### Visual Agnosia

Visual agnosia is a visuoperceptual disorder. It is defined as the inability to recognize objects presented visually, in the absence of any ocular or semantic deficit that could otherwise account for it (31). It is further divided into apperceptive and associative, according to the defective process being in the perceptual analysis of the object or in attributing a meaning to it, respectively. In PCA, the apperceptive subtype predominates (16, 32), demonstrated by the patient failing to copy a figure or match a figure with a sample (33). A particular form of visual agnosia affects the recognition of faces (prosopagnosia), a deficit that is a source of great social embarrassment. As with global visual agnosia, prosopagnosia in PCA is thought to be perceptual rather than agnosic in nature (17).

### Reading Disturbance

Trouble reading is one of the most frequent and disabling deficits for which PCA patients seek help. It can be due to acquired primary alexia (34), but most often reading impairment in PCA results from a combination of deficits including simultanagnosia, ocular apraxia, visual crowding (16, 24) and potentially homonymous visual field defects.

### Visual Field Defects

**Figure 1:**
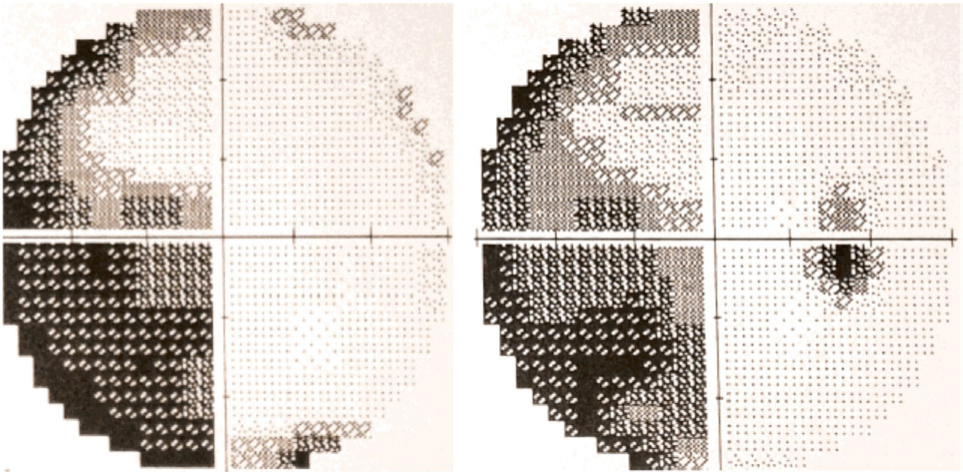
30-2 Humphrey automated perimetry from a patient with posterior cortical atrophy (PCA) depicting left incomplete homonymous hemianopia. Published with the patient’s authorization.

The occurrence of visual field defects in PCA has been a controversial subject, mainly due to the eloquence of the higher order visuospatial deficits that may arguably compromise the interpretation of visual field tests (35). Indeed, early PCA series dismissed visual field defects as exceptional in this condition (15). However, homonymous hemianopia or quadrantanopia was found in almost 50% of the patients in another series (7) and prevalence is even higher in groups of PCA patients who have visual fields performed as part of the workup (36–38), suggesting this deficit may be overlooked if not routinely searched for. Homonymous visual field defects are increasingly recognized as an early sign in PCA (39), and their occurrence may, remarkably, precede the higher order visual disorder (40, 41). Figure 1 shows a typical visual field test result in a patient with PCA. The inferior quadrants are possibly more affected in the visual variant of AD (4, 42); this would imply involvement of the underlying optic radiations as occurs with bilateral occipitoparietal infarction, but this needs confirmation.

### Other Visual Deficits

Besides these major, well-characterized deficits, patients with PCA have been reported to complain of a variety of visual problems, including perceived motion of static stimuli, visual crowding, color washout, and prolonged color afterimages (23, 24). In addition, related deficits previously documented in AD patients and attributed to visual cortical pathology, e.g., abnormal contrast sensitivity and loss of color discrimination, particularly affecting short wave length (blue) stimuli (43), may apply to PCA as well. Basic visual skills, such as form detection and discrimination, color perception, and motion coherence were more recently highlighted as possibly contributing to the higher order visual deficits classically reported in PCA (19). A specific dysfunction of the magnocellular (M) pathway in AD, associated with impairment of motion perception and loss of achromatic contrast, has been proposed (44).

### Non-Visual Deficits

Non-visual deficits in PCA are mainly represented by disturbances of numeracy and literacy. These deficits may occur as part of a Gerstmann syndrome (agraphia, acalculia, left-right disorientation, and finger agnosia) or may be isolated. Attentional disorders may occur as visual (16, 45) or spatial neglect (46) or a combination of the two. Ideomotor apraxia is not rare, but it is usually mild in early stages, and if prominent should raise the suspicion of an alternative diagnosis, such as CBD. The same can be said if features of asymmetric parkinsonism are found. Poly-mini-myoclonus has been suggested to be an overlooked sign in PCA, present in a majority of patients (17). Visual hallucinations and REM-sleep behavior disorder have been occasionally observed in PCA, and are also thought to indicate an underlying non-AD pathology, namely DLB (17). Depression is common and is thought to be mainly reactional (35).

It should be noted that dressing and constructional apraxias, which are very common in PCA (7, 47), do not constitute apraxia in its proper definition, but rather visuospatial deficits (48).

## Neural Correlates of the Visual Deficits in PCA

The higher order visual deficits observed in PCA patients are better understood as reflecting regional disruption of the two visual pathways for processing of object (ventral, “what” stream) and space (dorsal, “where” stream) (49, 50). This understanding has been favored by imaging studies showing correlation of simultanagnosia, the prototype of the visuospatial disorder in PCA, with greater atrophy in the dorsal (occipitoparietal) regions (20), while visuoperceptual deficits, e.g., visual agnosia, are associated with predominant volume loss in the ventral (occipitotemporal) regions (34). Based on this schematic representation of visual processing, a clinical classification of PCA into a dorsal and a ventral subtype has been proposed (16). The first subtype would be represented by patients with predominant Bálint syndrome, apraxia, and neglect, while the second would be characterized by disproportionate visual agnosia, prosopagnosia, and alexia. A third subtype has been suggested to include patients with primary visual failure and impairment in basic visual skills (9, 51), in whom more marked occipital damage would be expected. Indeed, homonymous hemianopia was recently found to be associated with lateralized occipital degeneration in PCA (38). Interestingly, however, even in the presence of visual field deficits, the primary visual cortex remains relatively unaffected compared to higher visual areas.

In clinical practice, most patients present with a confluence of deficits relating to the occipital, parietal, and posterior temporal lobes, and this is mirrored by imaging studies showing considerable overlap of patterns of atrophy when these subgroups of patients are analyzed in combination (20).

## Diagnosis

The critical element on the diagnosis of PCA clinico-radiological syndrome is the recognition of a progressive focal posterior cortical dysfunction associated with imaging evidence of damage to posterior cortical regions. As part of the clinical characterization, a formal neuropsychological assessment is necessary to establish the degree of involvement of individual cognitive domains and confirm that the disorder is relatively restricted to occipital and parietal regions.

### Diagnostic Criteria

Several clinical criteria for PCA have been published (7, 15, 16, 18), which are highly consistent in their definition of PCA. They all emphasize a higher visual disorder of insidious onset, manifesting with deficits of the dorsal and/or ventral stream, and which occurs with relative preservation of more anterior functions, such as memory and language. Some variability exists though in the delimitation of the syndrome. Remarkably, the Tang-Wai criteria (Box 1) introduce visual field defects as a core feature of PCA, given the same importance as simultanagnosia, constructional dyspraxia, environmental disorientation, and any element of the Gerstmann syndrome; at the same time, early parkinsonism and visual hallucinations, meant to distinguish LBD—a disease that may present with posterior cortical deficits but usually shows concomitant or fast developing involvement of other cortical and subcortical regions—are deemed exclusion criteria. Such phenotypic refinement may understandably impact on the specificity of these criteria.

Box 1 **Diagnostic criteria for posterior cortical atrophy Tang-Wai et al. (7).**

**Core features**

Insidious onset and gradual progression

Presentation of visual complaints in the absence of significant primary ocular disease explaining the symptoms

Relative preservation of anterograde memory and insight early in the disorder

Disabling visual impairment throughout the disorder

Absence of stroke or tumor

Absence of early parkinsonism and hallucinations

Any of the following findings:

 Simultanagnosia with or without optic ataxia or ocular apraxia

 Constructional dyspraxia

 Visual field defect

 Environmental disorientation

 Any of the elements of Gerstmann syndrome

**Supportive features**

Alexia

Presenile onset

Ideomotor or dressing apraxia

Prosopagnosia

**Investigations**

Neuropsychological deficits referable to parietal and/or occipital regions

Focal or asymmetric atrophy in parietal and/or occipital regions on structural imaging

Focal or asymmetric hypoperfusion/hypometabolism in parietal and/or occipital regions on functional imaging

Indeed, by excluding patients with specific LBD features, Tang-Wai et al. aimed to rule out LBD as a distinct condition from PCA; however, thereby, PCA caused by LBD or mixed (LBD-AD) pathologies are likely to be excluded as well (6, 7). Another conflicting issue is the inclusion of patients with PCA due CBD. Asymmetric parkinsonism and apraxia are suggested to distinguish PCA-CBD from PCA-AD, but these features are not addressed in a structured manner. In fact, if parkinsonism develops early in the course of disease, patients with PCA-CBD will be excluded. Therefore, while these criteria were not designed for the etiological diagnosis of PCA, they may be more specific for AD. On one hand, this characteristic has been critical for the very recognition of PCA as a variant of AD, on the other hand, a vacuum is left on how to classify those patients with a progressive posterior cortical dysfunction whose clinical features extend beyond the PCA typical phenotype.

**Figure 2:**
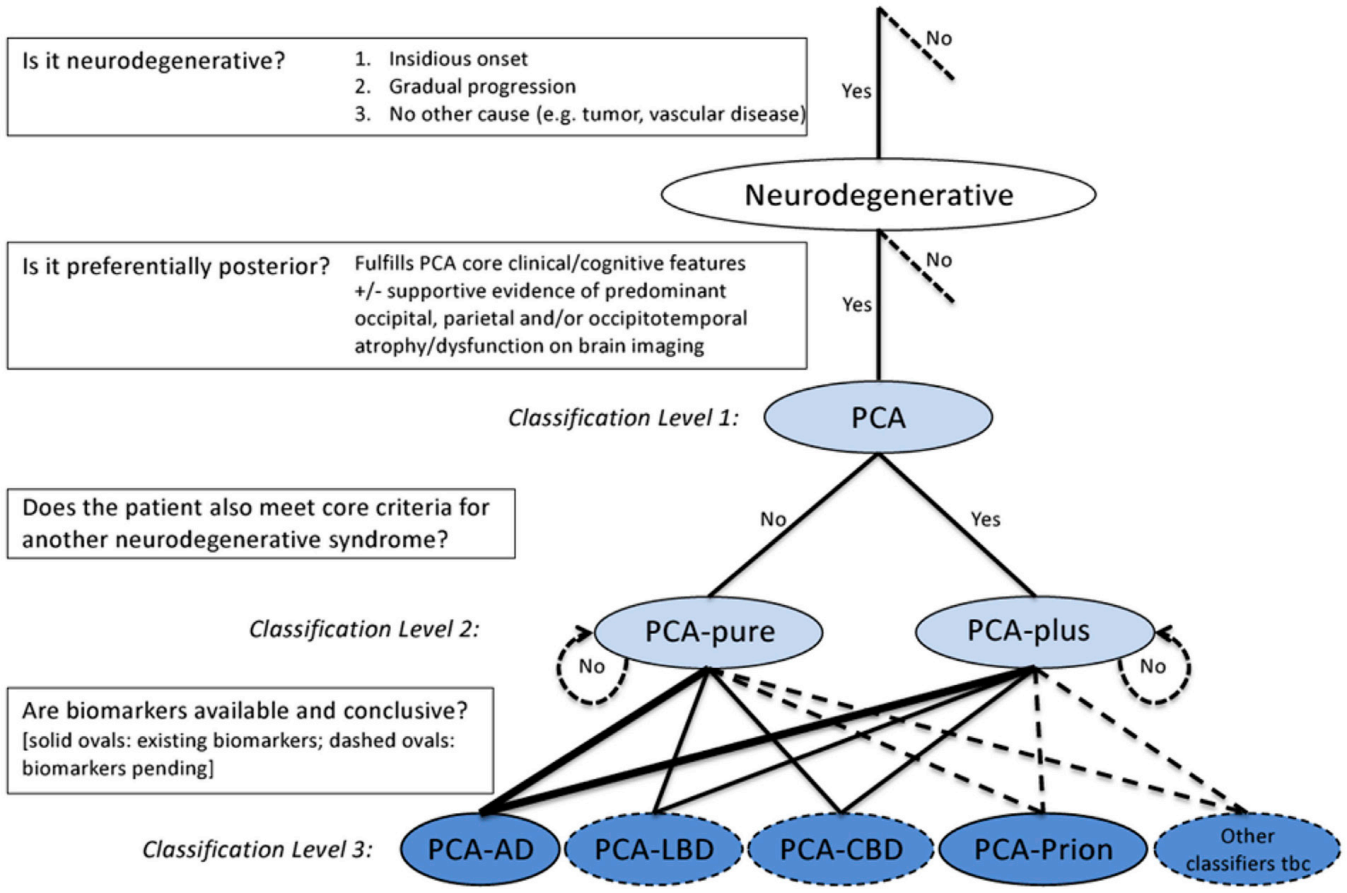
Diagnostic process and PCA classification. Key diagnostic questions at each level are shown in boxes. Syndrome-level descriptions (classification levels 1 and 2) are lightly shaded and disease-level descriptions (classification level 3) are darkly shaded. Among the disease-level classifications, PCA-AD and PCA-prion (solid ovals) are distinguished from PCA-LBD and PCA-CBD (dashed ovals) owing to the current availability of *in vivo* pathophysiological biomarkers. Other disease-level classifications may be appropriate (e.g., a patient with PCA plus visual hallucinations may have LBD-variant of AD) or anticipated (e.g., PCA attributable to GRN mutations). The thickness of lines connecting classification levels 2 and 3 is intended to reflect the status of AD as the most common cause of PCA. Abbreviations: AD, Alzheimer’s disease; CBD, corticobasal degeneration; LBD, Lewy body disease; PCA, posterior cortical atrophy; tbc, to be confirmed. Reproduced from Crutch et al. (52), available under the terms of Creative Commons Attribution License (CC BY 4.0).

A different (lumpers) approach to the diagnosis of PCA has recently been suggested (52). While preserving the core clinical description of the primary PCA syndrome (Box 2), the newly developed classification provides a solution on how to deal with atypical features, including them into a stratified framework to the etiological diagnosis (Figure 2): at the first level, the presence of the PCA clinico-radiological syndrome is established; the second level consists in of deciding whether PCA occurs in isolation (PCA-pure) or whether criteria for another neurodegenerative condition (e.g., visual hallucinations for LBD) are also met (PCA-plus); at the third level, these phenotypical categories are combined with the presence of pathology-specific biomarkers to yield a disease-level PCA description. Ideally, at this level, the patient will be given a diagnosis of PCA-AD, PCA-LBD, PCA-CBD, PCA-prion, and others. If a patient has PCA-pure and positive biomarkers for AD, the definitive diagnosis of PCA-AD may be given *in vivo*. However, since disease biomarkers are available only for AD and prion, and AD may manifest with such diverse phenotypes, diagnosis of PCA-LBD and PCA-CBD are currently presumptive and dependent on negativity to AD biomarkers. For instance, when patients fulfill criteria for both PCA and CBD and AD biomarkers are negative, the diagnosis of probable PCA-CBD may be appropriate. Alternatively, if a patient with PCA-plus tests positive for AD biomarkers, the diagnosis of AD may be given, although as disease-specific markers are not available for the second clinical suspected condition, the possibility of a dual pathology is still reasonable. These criteria take into account the suggested diagnostic criteria for AD (10) in their comprehensive approach of phenotype and disease-specific biomarkers. Their novelty is to include under the term PCA patients with posterior cortical dysfunction whom would otherwise not be given a unified diagnosis. Only further pathological studies will confirm the accuracy of these criteria. An expected consequence is an increment in the proportion of patients with non-AD pathologies in PCA cohorts. In the clinic the major priority is to recognize the syndrome and manage appropriately whatever the underlying pathology might be. Predictors of whether AD is the underlying pathology or not will not affect management materially until features of the other conditions are clinically apparent.

Box 2 **Core features of the posterior cortical atrophy clinicoradiological syndrome Crutch et al. (52).**

**Clinical features:**

Insidious onset

Gradual progression

Prominent early disturbance of visual ± other posterior cognitive functions

**Cognitive features:**

At least three of the following must be present as early or presenting features ± evidence of their impact on activities of daily living:

 Space perception deficit

 Simultanagnosia

 Object perception deficit

 Constructional dyspraxia

 Environmental agnosia

 Oculomotor apraxia

 Dressing apraxia

 Optic ataxia

 Alexia

 Left/right disorientation

 Acalculia

 Limb apraxia (not limb-kinetic)

 Apperceptive prosopagnosia

 Agraphia

 Homonymous visual field defect

 Finger agnosia

**All the following must be evident:**

 Relatively spared anterograde memory function

 Relatively spared speech and non-visual language functions

 Relatively spared executive functions

 Relatively spared behavior and personality

**Neuroimaging:**

Predominant occipitoparietal or occipitotemporal atrophy/hypometabolism/hypoperfusion on magnetic resonance imaging/^18^F-labeled fluorodeoxyglucose positron emission tomography/SPECT, single-photon emission computed tomography.

**Exclusion criteria:**

Evidence or a brain tumor or other mass lesion sufficient to explain the symptoms

Evidence of significant vascular disease including focal stroke sufficient to explain the symptoms

Evidence of afferent visual cause (e.g., optic nerve, chiasm, or tract)

Evidence of other identifiable causes for cognitive impairment (e.g., renal failure)

In the following sections, the imaging studies that support PCA as a clinico-radiological syndrome are reviewed, as well as *in vivo* pathological biomarkers for AD. In the particular case of LBD, to which no disease-specific biomarker is available, metabolic studies that are associated with the disease are also mentioned.

## Imaging Studies

### Magnetic Resonance Imaging (MRI)

**Figure 3:**
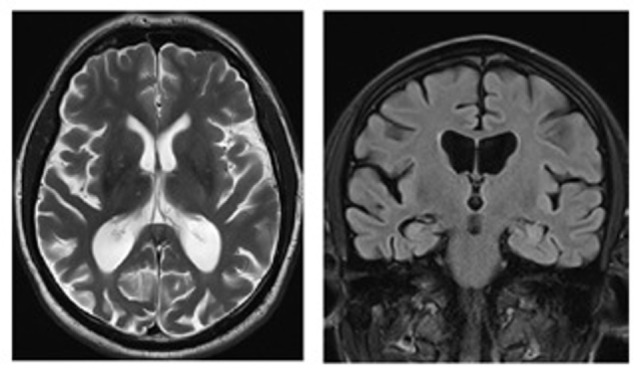
Magnetic resonance images (axial T2, coronal FLAIR) of a patient with posterior cortical atrophy demonstrating marked regional atrophy in the occipitotemporal regions and relative preservation of the hippocampi. Patient under the care of GTP; published with patient’s authorization.

**Figure 4:**
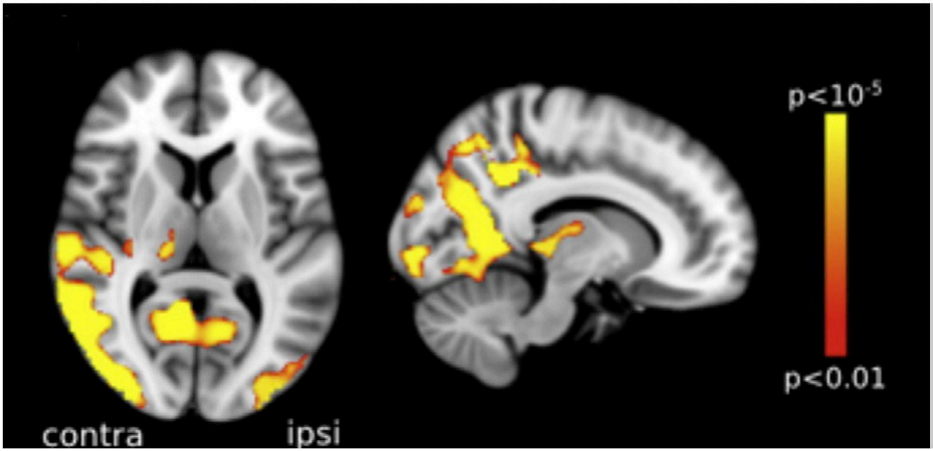
Voxel-based morphometry analysis comparing a group of posterior cortical atrophy (PCA) patients and age-matched healthy controls. Areas with most significant atrophy (highlighted in red-yellow) in PCA patients included the lateral and anterior occipital cortex, with some loss also noted in the parietal lobe, more marked in the hemisphere contralateral to the visual field defect, here represented on the left. Reproduced from Millington et al. (38), available under the terms of the Creative Commons Attribution License (https://creativecommons.org/licenses/by/4.0/).

The syndrome of PCA can manifest without any detectable gray or white matter volume loss at MRI (24), but more commonly patients present with marked atrophy in the occpitoparietal and occipitotemporal regions bilaterally, but often more severe in the right hemisphere (15, 53, 54) (Figure 3). Although the disparate posterior volume loss is used to distinguish PCA from typical AD at imaging, it is not rare for patients with PCA to present coexisting atrophy in the mesial temporal regions, including the hippocampi (54). However, when directly compared, studies have generally shown only subtle differences in levels of atrophy between PCA and AD, with PCA patients showing greater atrophy in the right visual association cortex, while left hippocampal atrophy predominates in amnestic AD (53, 54). Millington et al. (38) used multimodal MR imaging to investigate structural and functional brain changes in a cohort of patients with PCA, all with visual field defects. Compared with healthy controls, cortical activation was reduced in the occipital lobes, with no significant lateralization, while gray matter loss was greater in extrastriate occipital regions, but more marked in the hemisphere contralateral to the visual field deficit (Figure 4). Likewise, reduction in white matter integrity, which was widespread, was lateralized to the hemisphere originating the hemianopia but in the occipital lobes only.

### Positron Emission Tomography with Fluorodeoxyglucose (FDG-PET)

Functional imaging is used as evidence of neurodegeneration in PCA, being particularly useful when MRI is considered normal. When examined with FDG-PET, patients with PCA show hypometabolism that is more marked in the occipitoparietal regions, sometimes with involvement of the frontal eye fields, but with relative sparing of the mesial regions of the frontal and temporal lobes (55–57). Directly compared with amnestic AD, PCA patients present more severe occipitoparietal and/or occipital hypometabolism in the right hemisphere (47, 55, 57, 58).

Positron emission tomography with fluorodeoxyglucose has also been studied in the differential diagnosis between PCA and LBD, with variable results (57, 59). A common problem is that, in line with overlapping posterior presentations, occipital hypometabolism may occur in both conditions (7, 60). In a comparative study, patients with PCA showed greater hypometabolism in the right temporooccipital cortex, while LBD was distinguished by hypometabolism predominating in the left occipital cortex (57). In another study (59), however, rather than lateralization, it was the degree of asymmetry and anterior extension that best distinguished them: PCA and LBD were both associated with bilateral occipitoparietal hypometabolism, but a higher degree of asymmetry favored PCA, while extension to orbitofrontal and anterior temporal regions was suggestive of LBD. The relative preservation of the posterior cingulate cortex compared with the precuneus and cuneus—a sign that has shown to distinguish LBD from typical AD (61, 62)—was not specific to LBD when compared with PCA (59).

### PET Imaging of Microglia

This novel imaging modality is based on the involvement of microglia activation in the pathogenesis of neurodegenerative diseases. The PET tracer ^11^C-PBR28 binds to the translocator protein-18 kDa, which is overexpressed in activated microglia; therefore, its regional distribution can be interpreted as an effect of local degeneration (63). In ^11^C-PBR28-PET, patients with PCA showed greater binding in occipital, posterior parietal, and temporal regions compared to controls and were distinguished from amnestic AD patients by higher binding in the occipital cortex bilaterally (64). Despite these encouraging results, TPSO PET imaging still has some limitations, including the existence of non-binders (65).

### Dopamine Transporter Imaging

Albeit not part of PCA routine assessment, this test may be very helpful when the clinical diagnosis of LBD is in the differential, as well as in patients with established PCA who progress with LBD features, e.g., parkinsonism and visual hallucinations. The finding of low dopamine transporter concentration in basal ganglia, measured with PET or single photon emission tomography (SPECT), supports the diagnosis of LBD (60).

## Electrophysiology Studies

The study of event-related potentials (ERPs) involves the quantification of eletroencephalographic changes caused by cognitive, motor, or sensory stimulation. The responses are stereotyped and time-locked with stimulus, permitting analysis of the various stages of the neural process. The best recognized finding of ERP in AD patients is an abnormal P3b (also known as P-300), a higher cortical response associated with an update in working memory (66). Potentials associated with earlier visual processing, such as P1 and N1, may also be abnormal (67). The latter is ascribed to dysfunction of the visual association cortex (67), which is commonly involved by AD pathology (68). Interestingly, in non-symptomatic PSEN1 carriers (preclinical AD) tested with a visual recognition memory task, ERP were reduced in frontal and increased in occipital regions, suggesting that posterior cortical decline in AD is preceded by increased reliance on these regions (69). There is no group study of the visually evoked potential (VEP) in PCA, but single-case reports show that they may be normal (70) or delayed (40). This is likely explained by variable disease stages and clinical presentations. Indeed, Mares et al. (71) studied a PCA patient with pure alexia, showing bilaterally absent N170—an ERP component associated with word reading and activation of the visual word form area—and concomitantly abnormal P1 (initially delayed in the left hemisphere only, later bilaterally), indicating that the early visual cortical dysfunction may have contributed to the reading disorder. Since visual ERPs depend on the integrity of the visual system, the finding of abnormal VEPs may favor the diagnosis of PCA in the appropriate clinical context, but there is no evidence to support their use in the differential diagnosis with amnestic AD.

## AD Biomarkers

### Positron Emission Tomography with an Amyloid-binding Tracer (PET-Amyloid)

Amyloid imaging has a role in the diagnosis of AD, more so in presenile patients—when greater deposition of β-amyloid is not expected—but has no use in distinguishing PCA from amnestic AD, as the same global pattern of deposition is seen (17, 72). This is no surprise, since in pathological studies of PCA, the anterior-posterior gradient refers to the disparate concentration of tau-derived neurofibrillary tangles in posterior regions, while the distribution of β-amyloid is diffuse (7).

### Cerebrospinal Fluid (CSF) Analysis with Measurement of AD Pathology Biomarkers

Cerebrospinal fluid examination is not required for the diagnosis of PCA, but this test is usually recommended in presenile dementia to exclude treatable causes. Furthermore, in the current diagnostic approach to AD, CSF biomarkers have a positive predictive value when they show low concentrations of amyloid β, increased total tau, and increased phospho-tau (10). As with the PET-amyloid, the application of these tests to PCA patients did not help to distinguish them from amnestic AD (35), although they have a place in the differential diagnosis of AD and alternative underlying pathologies.

### Tau-PET

Several tau-specific tracers for PET, including ^18^F-AV-1451 (Flortaucipir) were recently made available for clinical assessment of various tauopathies (73). Although their affinity for specific tau deposits is still to be established, the application of tau-PET to the diagnosis of AD is promising. Because ^18^F-AV-1451 binds to hyperphosphorylated paired helical filament tau and neurofibrillary tangles (73), and tau pathology—but not amyloid—is at disproportionate higher concentration in posterior brain regions in PCA (5, 7), the tau-PET has the additional potential to distinguish PCA from other AD variants. A pattern with localized elevation of ^18^F-AV-1451 to posterior regions has indeed been shown to strongly correlate with PCA (74, 75), distinguishing it from amnestic AD (76). Moreover, regional tau-binding mirrors regional patterns of both hypometabolism (74, 75) and atrophy (75) across AD major phenotypes, PCA included. For this reason, it is possible that this test be included as an *in vivo* imaging biomarker of AD in future (75).

### Serum Biomarkers of AD

Decrement of peripheral β-amyloid does occur in AD, but later than CSF levels, so that an important effect is observed at the stage of dementia only (77). Plasma levels of Aβ have not been studied in PCA. Markers of neuronal injury, the tau, and neurofilament light (NFL) proteins are elevated in the sera of AD patients, but levels significantly overlap with those of controls and individuals with mild cognitive impairment, although NFL may be more accurate (78). These difficulties have prevented the recognition of a serum signature of AD. Plasma NFL levels further correlate with longitudinal measures of cognition and atrophy in AD and have been suggested as a tool for screening patients at risk of cognitive decline (78); however, NFL is not specific for AD, thus cannot help in the differential diagnosis with other neurodegenerative conditions.

## PCA as a Phenotype within the Spectrum of AD Clinical Variability

The predominance of right hemisphere deficits in PCA patients observed in metabolic (47, 55, 57, 58, 79), as well as structural (20, 54, 80) imaging is intriguing. Some of the syndrome’s most characteristic deficits correlate with either bilateral (e.g., simultanagnosia) or dominant hemisphere (e.g., Gerstmann syndrome) dysfunction (18). Besides, a selective vulnerability of the right hemisphere cannot be easily hypothesized on a pathological basis. The explanation for such disparity may instead lie with the syndrome definition. Failure in visual object and space perception, which underlies the concept of PCA as a higher order visual disorder, is indeed associated with bilateral occipital and right parietal damage (74, 81). A left hemisphere dysfunction would manifest with predominant language deficits; accordingly, relatively focal left parietal atrophy/hypometabolism is a topographical marker of logopenic primary progressive aphasia (lvPPA) (82), the “language” variant of AD (10). In line with syndrome continuity, when patients with PCA develop language problems, these are usually dominated by word retrieval deficits, a clinical overlap with lvPPA (12).

In clinic, the differentiation between PCA and amnestic AD is more often a challenge, for the latter is common and can also present with visuospatial deficits. The basis for these common deficits is a shared neuroanatomic substrate that critically involves the parietal lobes. This has been identified as the “default model network” (DMN), after the observation that it activates during non-focused rest, which encompasses structures such as the medial-temporal lobe, precuneus, posterior cingulate, and temporoparietal junction (83), with a central role suggested to the precuneus (84). The DMN is commonly affected across AD variants, however at disproportionate, syndrome specific regional severities (11, 85). In typical AD, pathology starts in the entorhinal cortex, then stereotypically spreads to limbic regions then to isocortex, including parietal association cortex (68, 86). In PCA, the higher pathology burden is found in the primary visual and visual association cortices, with the posterior parietal regions being commonly involved as well (5). Likely reflecting pathology, at metabolic imaging, the highest degree of overlap between AD variants localizes to the dorsal DMN (85). From a dynamic perspective, the parietal lobe may thus be seen as a hub where PCA and AD meet, in diverging directions, within a common network of progression. Accordingly, patients with PCA show decreased functional connectivity in the visual network, in various regions—including the precuneus—within the DMN, as well as more anterior structures (87). In addition, white matter loss is more diffuse in PCA than would be expected from its relatively focal posterior presentation (38, 87), likely anticipating more anterior deficits. A consequence of these converging patterns of degeneration between PCA and typical AD is that the diagnosis of patients presenting with focal parietal deficits may be challenging. For instance, in a patient with a relative isolated visuoconstructive disorder and right parietal hypometabolism, the differential diagnosis between AD and PCA may not be accurate until further memory or visual deficits develop, and/or hypometabolism extends to more temporal or occipital regions, respectively, although the preservation of memory and the lateralized right presentation could increase the odds for PCA.

## Genetics

Posterior cortical atrophy is predominantly a sporadic condition. However, the PCA phenotype has rarely been described in association with genetic mutations known to be implicated in familial AD, [*PSEN1* (88, 89) and *PSEN2* (90)], mutations associated with frontotemporal dementia [*MAPT* (91) and *GRN* genes (8)], as well as in the gene of the prion protein (*PRNP*) (92).

In recent years, there has been an effort to understand the factors driving phenotypic variation in AD. Previous investigations of the commonest genetic risk factor to late onset AD, the allele ε4 of the *APOE* yielded conflicting results in PCA, with some studies suggesting that variation in this gene confers an increased risk to the visual variant of AD (54, 93), while no association was found by others (13, 94). A recently published consortium study, which included the largest number of PCA patients to date, reported a robust association between variation in/near *APOE*/*TOMM40* and risk for PCA, but with a smaller effect than that for amnestic AD (95).

Variants of *TREM2* and *PSEN2* that modify the risk for AD have also been reported in PCA (93), but there is no evidence for any particular effect in PCA as compared to amnestic AD.

## Treatment

No study is available reporting the effectiveness of acetyl-cholinesterase inhibitors in this condition. Nonetheless, given the strong pathological association with AD, most clinicians dealing with PCA patients find it appropriate to offer them a trial of these drugs. Likewise, memantine is sometimes tried in individual cases.

A considerable part of the management of PCA consists of assisting patients in taking decisions about their occupational and daily lives, considering that, albeit slowly, vision and cognition will continue to deteriorate. Among lifestyle changes stopping driving should be recommended. One of us (GTP) considers the condition to be appropriate grounds for registration as “severely sight impaired” which in the UK is equivalent to being registered as “legally blind” despite normal visual acuity in the early stages.

## Conclusion

Posterior cortical atrophy, a clinico-radiological syndrome that in most cases represents a focal form of AD, is unique among the known dementing conditions for causing a highly disabling visual disorder with preserved cognitive status in the early stages. The course of PCA due to AD is stereotyped with virtually all patients later developing memory loss and progressing into full dementia. The recognition of PCA as an atypical variant of AD and the availability of accurate AD biomarkers has made PCA a condition where a diagnosis of definitive AD can be given *in vivo*. When non-AD pathologies are the cause of PCA, clues for the alternative pathology are often found in clinical features as well as imaging.

Despite the progress in the understanding of the neural basis of PCA in recent years, patients still frequently experience a painful delay in diagnosis, mainly because it is not appreciated that their symptoms are associated with brain dysfunction by optometrists and ophthalmologists who are consulted. The need of increasing awareness among clinicians cannot be overestimated, and this should involve not only neurologists, but general practitioners, optometrists, and ophthalmologists.

For all who see patients with visual symptoms we would emphasize the following. First, in the anamnesis, take note of visual symptoms that have an emphasis on spatial disorientation. Second, in the basic clinical assessment, such features as unexplained difficulty with Ishihara plates, variable homonymous defects on perimetry and a tendency to omit letters on the acuity chart should raise suspicion of the disorder.

## Author Contributions

GP conceived project and revised early drafts of the article. HB, RM, and MJ-G revised early drafts of the article. MNMdS generated first draft of article.

## Conflict of Interest Statement

The authors declare that the research was conducted in the absence of any commercial or financial relationships that could be construed as a potential conflict of interest.
